# Dobinin K Displays Antiplasmodial Activity through Disruption of *Plasmodium falciparum* Mitochondria and Generation of Reactive Oxygen Species

**DOI:** 10.3390/molecules29194759

**Published:** 2024-10-08

**Authors:** He Sun, Bo-Chao Liu, Long-Fei He, Chao-Jiang Xiao, Bei Jiang, Lei Shen

**Affiliations:** 1 Yunnan Key Laboratory of Screening and Research on Anti-pathogenic Plant Resources from Western Yunnan, Dali University, Dali 671000, China; 2 Institute of Materia Medica, Dali University, Dali 671000, China; 3 College of Pharmacy, Dali University, Dali 671000, China

**Keywords:** dobinin K, *Plasmodium*, reactive oxygen species, mitochondria

## Abstract

Dobinin K is a novel eudesmane sesquiterpenoids compound isolated from the root of *Dobinea delavayi* and displays potential antiplasmodial activity in vivo. Here, we evaluate the antiplasmodial activity of dobinin K in vitro and study its acting mechanism. The antiplasmodial activity of dobinin K in vitro was evaluated by concentration-, time-dependent, and stage-specific parasite inhibition assay. The potential target of dobinin K on *Plasmodium falciparum* was predicted by transcriptome analysis. Apoptosis of *P. falciparum* was detected by Giemsa, Hoechst 33258, and TUNEL staining assay. The reactive oxygen species (ROS) level, oxygen consumption, and mitochondrial membrane potential of *P. falciparum* were assessed by DCFH-DA, R01, and JC-1 fluorescent dye, respectively. The effect of dobinin K on the mitochondrial electron transport chain (ETC) was investigated by enzyme activity analysis and the binding abilities of dobinin K with different enzymes were learned by molecular docking. Dobinin K inhibited the growth of *P. falciparum* in a concentration-, time-dependent, and stage-specific manner. The predicted mechanism of dobinin K was related to the redox system of *P. falciparum*. Dobinin K increased intracellular ROS levels of *P. falciparum* and induced their apoptosis. After dobinin K treatment, *P. falciparum* mitochondria lost their function, which was presented as decreased oxygen consumption and depolarization of the membrane potential. Among five dehydrogenases in *P. falciparum* ETC, dobinin K displayed the best inhibitory power on NDH2 activity. Our findings indicate that the antiplasmodial effect of dobinin K in vitro is mediated by the enhancement of the ROS level in *P. falciparum* and the disruption of its mitochondrial function.

## 1. Introduction

Malaria is an insect-borne infectious disease caused by the bite of *Anopheles* mosquitoes carrying the malaria parasite or by contact with the blood of an infected person [1]. An amount of 249 million estimated malaria infections caused 608,000 deaths globally in 2022. Both the incidence rate and mortality rate of malaria in 2022 are slightly increased compared to that in 2021. Moreover, 76.0% of total malaria deaths were children under 5 years old, which revealed that malaria remains a severe public health problem [2]. The symptoms of *Plasmodium falciparum* (*P. falciparum*), which is the deadliest *Plasmodium* species, can be cured by remarkably effective antimalarial drugs [3]. Unfortunately, more and more drug-resistant cases of *P. falciparum* have been reported recently. Additionally, even a resistance to artemisinin, which is the first-line antimalaria agent, has appeared [4,5,6]. Historically, natural products and their derivatives have been the preferred resources for the discovery of antimalarial active substances, which include quinine and artemisinin, as well as numerous second-generation drugs derived from them. However, once parasite resistance occurs against one of these drugs, a series of drugs will face the danger of ineffectiveness due to their similar structures and acting targets [7]. Therefore, it is urgent to pursue novel potential antimalarial lead compounds that, unlike classic agents, overcome the resistance of *Plasmodium*.

In virtue of the diversity of topography and climate, the western Yunnan province of China has rich plant resources, some of which still have unknown medicinal value. From our consistent effort of searching for antimalarial compounds from these resources, dobinin K, a novel eudesmane sesquiterpenoids compound isolated from the root of *Dobinea delavayi*, has been confirmed to possess potential antimalarial activities [8]. *Dobinea delavayi* is a perennial herb belonging to the Anacardiaceae family and is used as folk medicine in Yunnan to treat mumps, mastitis, and traumatic injury. The antimalarial activity of dobinin K against *Plasmodium yoelii* BY265RFP has been evaluated in vivo. The results showed that the inhibition ratio of 30 mg/kg dobinin K was 59.1%, which is stronger than that of artemisinin at a dose of 50 mg/kg (54.4%). Thus, it is necessary to further study the antimalarial potential in vitro and the acting mechanism of dobinin K [9].

Classic antimalarial drugs act on a few limited targets that are involved in various metabolic and biochemical pathways of *Plasmodium*, such as hemozoin synthesis, fatty acid biosynthesis, folate metabolism, etc. The mitochondria of *Plasmodium* are essential for the survival of parasites and have remarkable molecular and functional differences from the mitochondria of humans [10]. In *Plasmodium*, only a small amount of energy comes from ATP that is produced by oxidative phosphorylation from the tricarboxylic acid cycle in mitochondria, and the main energy supply relies on glycolysis that is carried out in the cytoplasm [11,12,13]. The major function of *Plasmodium* mitochondria is pyrimidine synthesis, which is entirely dependent on the de novo synthesis pathway without the rescue pathway [14,15]. These unique characteristics of this organelle make it become a potential target for antimalarial agents.

In this study, we have comprehensively evaluated the antiplasmodial activities of dobinin K on different *P. falciparum* strains, including chloroquine-sensitive strain (3D7), chloroquine-resistant strain (Dd2), and artemisinin-resistant strain (803). Then, based on the predicted results of transcriptome analysis, we explored the possible effects of dobinin K on *Plasmodium* mitochondria and the components of the electron transport chain (ETC). The results of this study demonstrate that dobinin K is a compound that could be used for the treatment of malaria.

## 2. Results

### 2.1. Dobinin K Is a Potent Antiplasmodial Compound In Vitro

**Figure 1:**
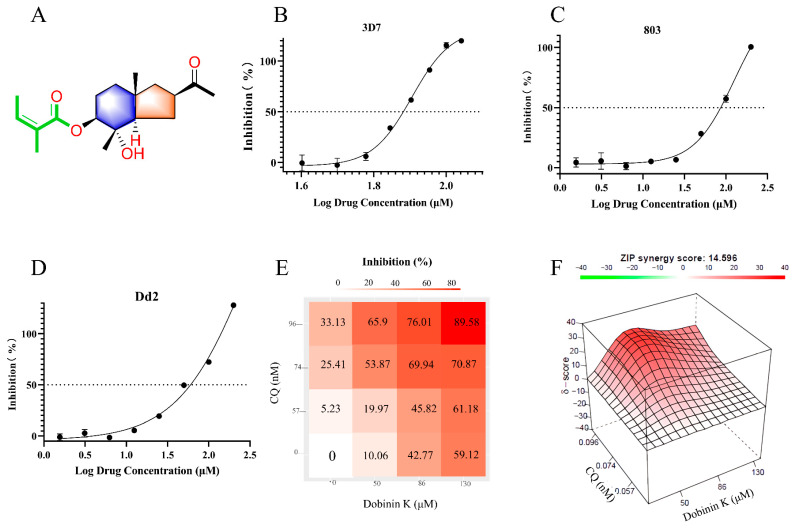
In vitro antiplasmodial activity of dobinin K against *P. falciparum*. (**A**) Chemical structure of dobinin K. (**B**–**D**) The fit curve showing parasitemia of 3D7 (**B**), 803 (**C**), and Dd2 (**D**) against logarithm concentration of dobinin K. (**E**,**F**) The synergistic potency between dobinin K and chloroquine against Dd2 was analyzed by the Zero interaction potency (ZIP) model. (**E**) This shows the schematics of the dose design. (**F**) This is a ZIP model result image that is displayed as a 3D heat map. CQ, chloroquine.

**Figure 2:**
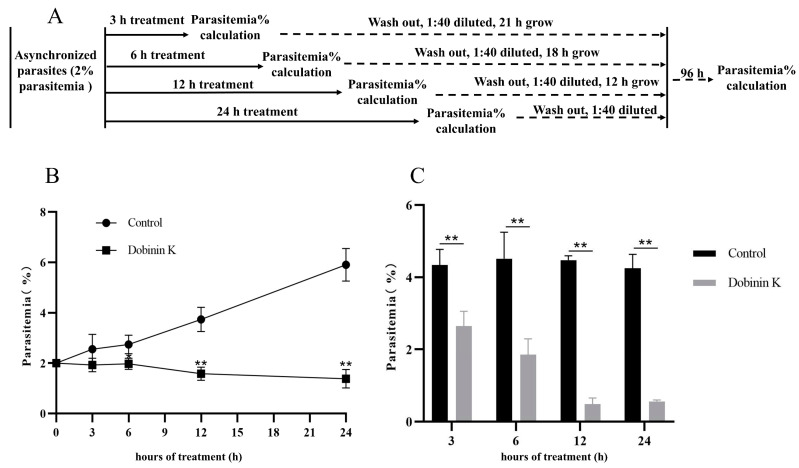
Time-dependent antiplasmodial activity of 100 μm dobinin K on 3D7 strain. (**A**) Schematics of experimental design. (**B**) The plot shows parasitemia against dobinin K incubating time. (**C**) The bar graph shows the long-term effect of dobinin K. The treated parasites from B were washed, followed by 1:40 dilution and 96 h culture. Data are presented as means ± SD (*n* = 3). All data come from 3000 RBCs. ** *p* < 0.01, compared to the control.

For evaluation of the antiplasmodial activity of dobinin K (chemical structure as shown in Figure 1A), chloroquine-sensitive strain (3D7), artemisinin-resistance strain (803), and chloroquine-resistance strain (Dd2) were applied. The inhibitory effects of dobinin K on these strains were indicated by the fluorescence intensity of SYBR Green I. After 72 h treatment, dobinin K showed a concentration-dependent antiplasmodial activity. The IC_50_ value of dobinin K against 3D7, 803, and Dd2 was 72.7 μM, 86.2 μM, and 65.2 μM, respectively (Figure 1B–D). Moreover, the activity of the combination of dobinin K and chloroquine against Dd2 was studied. The ZIP synergy score was 14.596 (>10), which displayed synergistic potency between the two drugs (Figure 1E,F). Asynchronous 3D7 was used to investigate time-dependence of the antiplasmodial activity of dobinin K. The results indicated that, compared to the control, the 12 h and 24 h treatments of dobinin K significantly suppressed *P. falciparum* growth (*p* < 0.01) (Figure 2B). For monitoring the long-term effect of dobinin K, the parasitemia was measured after a washout of dobinin K and 96 h culture. We found that all treatments of dobinin K (3, 6, 12, and 24 h) induced a substantial reduction in parasitemia (Figure 2C). The above results showed that dobinin K had an extent of antiplasmodial potency with concentration- and time-dependence.

### 2.2. Dobinin K Suppresses the Growth of P. falciparum at the Trophozoite and Schizont Stages

**Figure 3:**
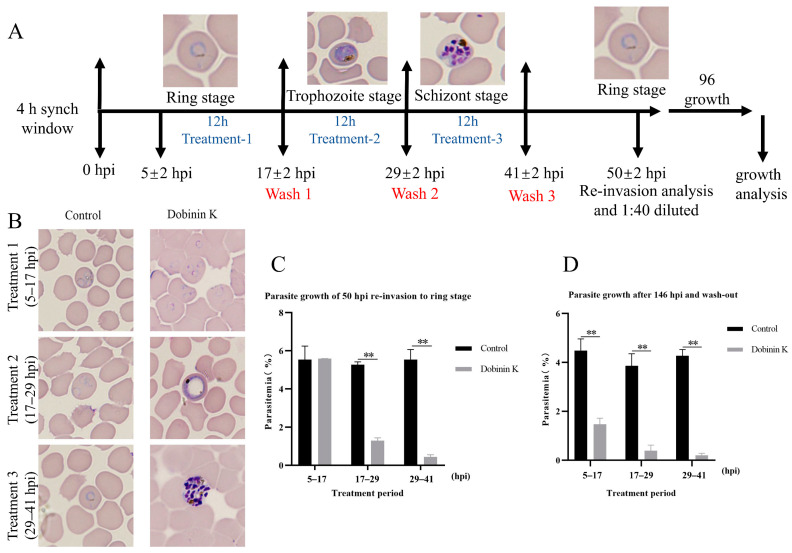
Stage-dependent antiplasmodial activity of dobinin K. (**A**) Schematics of experimental design. (**B**) Giemsa staining images showing different morphologies of parasites at 50 hpi after the dobinin K treatment. The treatment was executed at the ring, trophozoite, or schizont stage. (**C**) The bar graph shows parasitemia at 50 hpi after 12 h dobinin K treatment. (**D**) The bar graph showing the long-term effect (146 hpi) of dobinin K. The treated parasites at 50 hpi from C were washed, followed by 1:40 dilution and 96 h culture. Data are presented as means ± SD (*n* = 3). All data come from 3000 RBCs. hpi, hours post invasion. ** *p* < 0.01 compared to the control.

Highly synchronized 3D7 *P. falciparum* was used to evaluate the stage specificity of the inhibitory effect of dobinin K. Parasites were exposed to dobinin K (100 μM) for a 12 h treatment at different stages including the ring (5–7 hpi), trophozoite (17–29 hpi), and schizont (29–41 hpi) (Figure 3A). Then, morphological analysis was performed at 50 hpi (1 growth cycle), and parasitemia was calculated at 50 hpi and 146 hpi (three growth cycles). At 50 hpi, the dobinin K treatment at trophozoite and schizont stages could strongly limit the development of parasites to the next cycle with the comparison of the control, but the treatment at the ring stage showed no obvious inhibition of the growth of parasites (Figure 3B,C). However, at 146 hpi, dobinin K treatment at all blood stages displayed strong blockage of parasite development (Figure 3D).

### 2.3. Target Prediction of Dobinin K Based on Transcriptome Analysis

**Figure 4:**
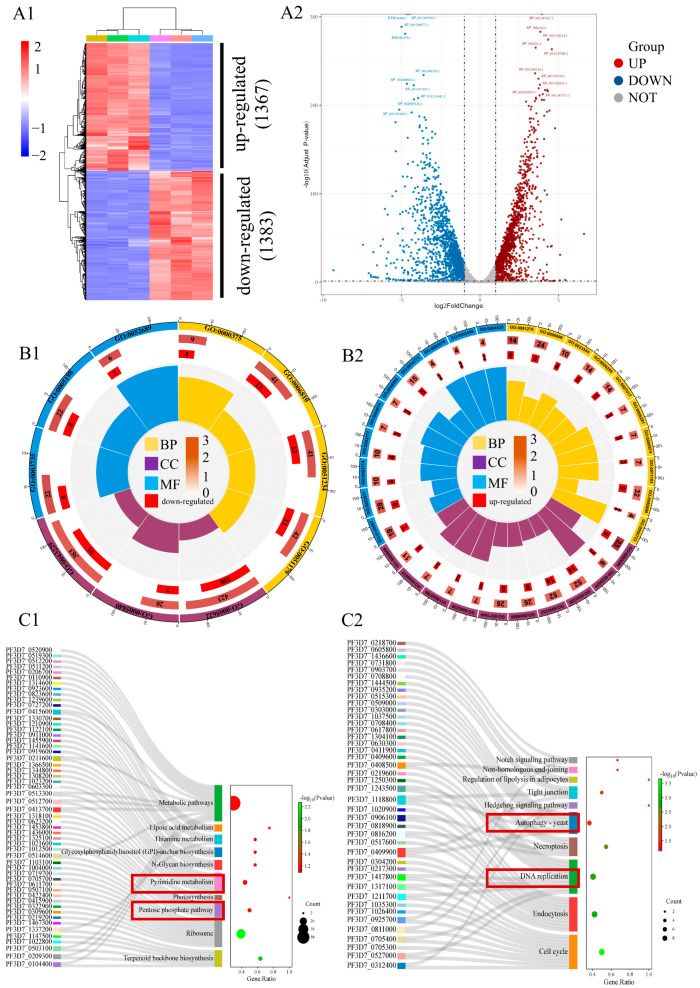
Transcriptome analysis of *P. falciparum* after the treatment of dobinin K. (**A**) The heatmap plot (**A1**) and the volcano plots (**A2**) showing *P. falciparum* genes significantly differed (*p* < 0.05) under the treated condition. The red grid and dot represent up-regulated genes (log FC > 1), the blue grid and dot represent down-regulated genes (log FC < −1), and the grey dot represents unchanged genes. (**B**) GO analysis based on differentially expressed genes of downregulation (**B1**) or upregulation (**B2**). Yellow, purple, and blue represent BP, CC, and MF, respectively. The first lap indicates the top 10 GO terms. The second lap indicates the number of genes in the genome background and *p*-values for gene enrichment for the specified GO terms. The third lap demonstrates a bar graph of enriched genes. The fourth lap exhibits the enrichment factor for each GO term. (**C**) KEGG analysis based on differentially expressed genes of downregulation (**C1**) or upregulation (**C2**). The sankey diagram is on the left, represents the genes contained in each pathway. The bubble map is on the right. The size of the bubbles indicates the number of genes involved in the signal pathway, and the color of bubble represents the *p*-value.

In order to capture global gene expression changes associated with dobinin K (73 μM, 12 h treatment), transcriptome analysis was performed. With the comparison of the control group and using a fold change cutoff of 2 and a *p*-value cutoff of 0.05, unsupervised hierarchical clustering analysis revealed that 2750 genes, of which 1367 were up-regulated and 1383 were down-regulated, were differentially expressed from the treatment group (Figure 4A).

The GO enrichment analysis of differentially expressed genes revealed the gene functional changes of 3D7 *Plasmodium* after the treatment with dobinin K. As the results of up-regulated genes showed, the biological processes (BP) focused on DNA metabolism and cellular responses to DNA damage stimuli. The cellular component (CC) was mainly enriched in organelles, nuclear chromosomes, and cytoskeleton components. The molecular functions (MF) mainly focus on the catalytic activity of DNA, double-stranded DNA binding, phosphotransferase activity, and cytoskeleton movement. The results of functional changes of down-regulated genes demonstrated that BP focused on the material transport process. CC was mainly enriched in organelles and ribosomes. MF was mainly focused on ribosome function and carboxylate hydrolase activity. Notably, most of the up-regulated differential genes were involved in the response to DNA damage in *P. falciparum*, which is largely caused by ROS (Figure 4B).

The KEGG analysis was used to reveal the signal pathways enriched by the differentially expressed genes. Up-regulated genes were mainly enriched in chromosome non-homologous end joining, autophagy, and necroptosis. Down-regulated genes were mainly enriched in metabolic pathways, pyrimidine metabolism, and the pentose phosphate pathway. Since pyrimidine metabolism is a major function of mitochondria and the pentose phosphate pathway is involved in reduction systems in *P. falciparum*, KEGG analysis results suggested that dobinin K may affect mitochondrial function and redox systems of *P. falciparum* (Figure 4C). The results of GO and KEGG analysis suggested that dobinin K may increase the ROS level, which is caused by damage of mitochondrial function, and destroy the pentose phosphate-pathway-associated reduction, then disrupt the redox homeostasis in *P. falciparum*, and finally induce oxidative damage.

### 2.4. Dobinin K Induces Apoptosis of P. falciparum and Increases Intracellular ROS Levels

**Figure 5:**
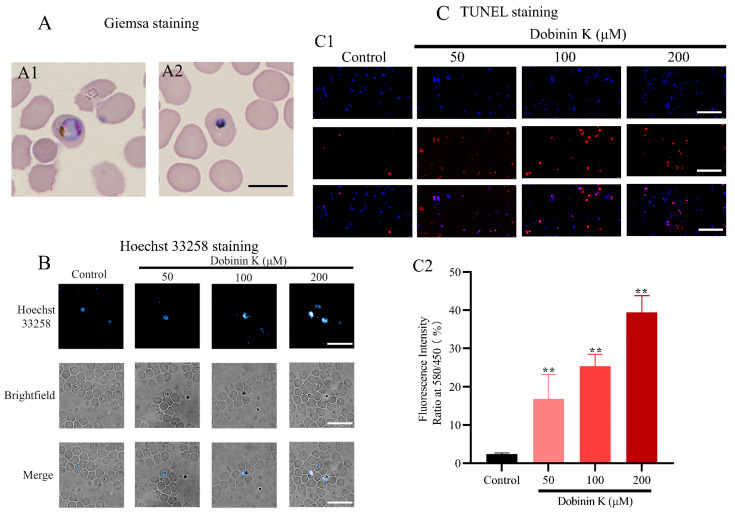
Dobinin K induces apoptosis of *P. falciparum*. (**A**) Apoptotic morphologies of *P. falciparum*, such as trophozoite stage untreated (**A1**), cytoplasm condensation, and deep nuclear staining (**A2**), are observed by Giemsa staining after dobinin K treatment. Bar = 5 μm. (**B**) Apoptosis of *P. falciparum* is observed by Hoechst 33258 fluorescent dying. Bar = 20 μm. (**C**) Apoptosis of *P. falciparum* is observed by TUNEL staining. (**C1**). Fluorescence microscope images of the nucleus (blue, DAPI staining) and DNA fragmentation (red, TUNEL staining). Bar = 20 μm. (**C2**) The bar graph shows the fluorescence intensity of TUNEL staining with the treatment of different concentrations of dobinin K. Data are presented as means ± SD (*n* = 3). ** *p* < 0.01 compared to the control.

Apoptosis of *P. falciparum* was observed by Giemsa staining, Hoechst 33258 fluorescent dying, and TUNEL staining after 12 h treatment of dobinin K. The results of Giemsa-stained smears exhibited morphologic abnormalities in the compound-treated group, including deep nuclear staining and cytoplasm condensation (Figure 5A1,A2). A similar apoptosis was seen in Hoechst 33258-stained cells after dobinin K incubation. These cells produced apoptotic bodies and underwent chromatin condensation, which were stained with higher blue fluorescence (Figure 5B). The degree of DNA fragmentation, which is one marker of apoptosis, was evaluated by fluorescence microscopy after TUNEL staining (Figure 5C1). Compared with the control group, dobinin K increased the proportion of red fluorescence with concentration dependence, and the proportions of TUNEL-positive cells in 50, 100, and 200 μM dobinin K groups were 16.84%, 25.30%, and 39.40%, respectively (Figure 5C2).

**Figure 6:**
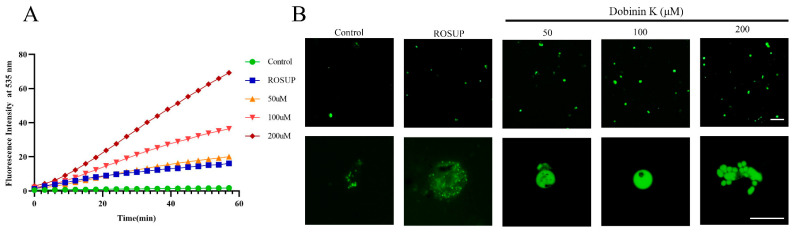
Dobinin K promotes intracellular ROS generation. (**A**) The plots showing ROS levels in *P. falciparum* against exposure time of dobinin K. ROS level is presented by DCFDA fluorescence intensity. (**B**) Fluorescence images are collected by fluorescence microscope under the treatment of different concentrations of dobinin K. Bar = 20 μm.

Intracellular ROS production was measured by DCFDA staining, and ROSUP was applied as a positive control. Compared to the untreated control, dobinin K (50, 100, 200 μM) treatment significantly increased the ROS level during 60 min with time-dependence, moreover, the abilities of all concentrations of dobinin K were stronger than ROSUP (Figure 6A,B).

### 2.5. Dobinin K Decreases Oxygen Consumption and Disrupts Membrane Potential (Δψm) of P. falciparum Mitochondria

**Figure 7:**
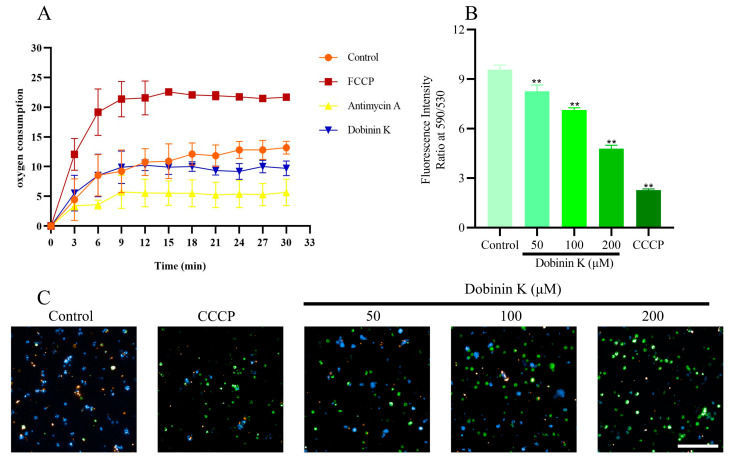
Dobinin K decreases oxygen consumption and disrupts membrane potential (Δψm) of *P. falciparum* mitochondria. (**A**) The plot shows the oxygen consumption against the exposure time of dobinin K. Oxygen consumption is presented by R01 fluorescent intensity. (**B**,**C**) Δψm of *P. falciparum* mitochondria is measured by JC-1 fluorescent dye. (**B**) The bar graph shows the fluorescence intensity ratio at 590/530. Data are presented as means ± SD (*n* = 3). ** *p* < 0.01 compared to control. (**C**) Fluorescence microscope images of the nucleus (blue, DAPI staining), normal membrane potential (red, JC-1 polymer staining) and damaged membrane potential (green, JC-1 monomer staining). Bar = 20 μm.

Isolated *P. falciparum* mitochondria were prepared to study the effect of dobinin K on oxygen consumption and membrane potential. 30 min treatment of 100 μM dobinin K resulted in a decrease in mitochondrial oxygen consumption. By comparison, FCCP, a mitochondrial oxidative phosphoric acid uncoupling agent, caused an increase in oxygen consumption, and antimycin A, a respiratory chain inhibitor, reduced oxygen consumption (Figure 7A). These results suggested that dobinin K may act on the mitochondrial respiratory chain.

A sensitive fluorescent dye, JC-1, was applied to measure Δψm. In the normal mitochondria, JC-1 formed an aggregate in the mitochondrial matrix and emitted a red fluorescence at 590 nm, but after the damage of mitochondria, JC-1 presented as a monomer and emitted a green fluorescence at 530 nm. In the untreated control group, the mitochondria showed a red stain of JC-1, but in the dobinin K-treated and CCCP-treated (positive control) groups, the mitochondria presented a green stain. The ratio of fluorescence intensity at 590/530 was 8.26 ± 0.38, 7.13 ± 0.13, and 4.78 ± 0.21 in 50 μM, 100 μM, and 200 μM dobinin K-treated cells, respectively. The ratio was 9.58 ± 0.28 in the case of untreated control and 2.27 ± 0.07 in the CCCP-treated cells (Figure 7B,C).

### 2.6. Dobinin K Effectively Inhibits P. falciparum NDH2 Activity

**Figure 8:**
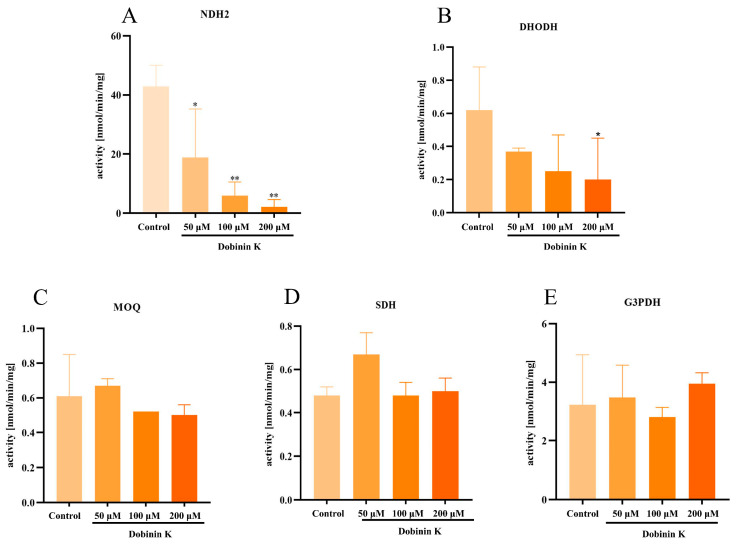
Effects of dobinin K on activities of five ETC dehydrogenases (**A**) NDH2, (**B**) DHODH, (**C**) MOQ, (**D**) SDH, (**E**) G3PDH) in *P. falciparum* mitochondria. Data are presented as means ± SD (*n* = 3). ** *p* < 0.01, * *p* < 0.05 compared to the control.

The effect of dobinin K at the concentrations of 50, 100, and 200 µM on five mitochondrial dehydrogenases of ETC was evaluated. The results showed that dobinin K significantly inhibited NDH2 activity (Figure 8A), and weakly depressed DHODH activity (Figure 8B), but could not affect the activities of MQO, SDH, and G3PDH (Figure 8C–E). The activities of NDH2 in the untreated control, 50 μM, 100 μM, and 200 μM dobinin K group were 42.87 ± 7.15, 18.81 ± 16.44, 5.65 ± 4.60 and 2.14 ± 2.48 U/mg prot, respectively. The activities of DHODH in the untreated control, 50 μM, 100 μM, and 200 μM dobinin K group were 0.62 ± 0.26, 0.37 ± 0.02, 0.25 ± 0.22, and 0.20 ± 0.25 U/mg prot, respectively.

### 2.7. Molecular Docking Study of Dobinin K with PfNDH2 and PfDHODH

**Figure 9:**
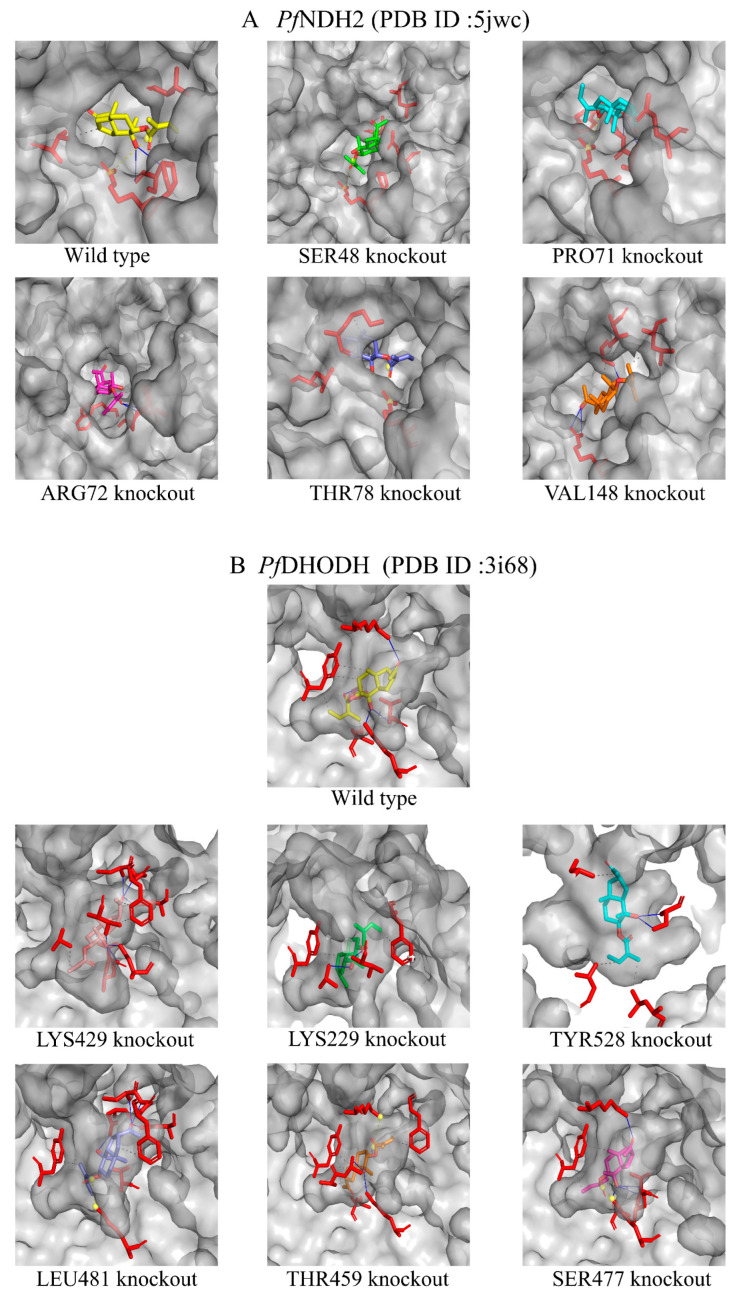
Molecular docking study of dobinin K with *Pf*NDH2 (**A**) and *Pf*DHODH (**B**). Each figure included molecular docking representation of the interaction between dobinin K and wild type protein, and that of the interaction between dobinin K and mutant protein with theoretical knockout of different residues. The red bar represents residues of protein, all other color bars represent dobinin K; each dobinin K color represents each docking situation after one residue knockout.

The results of molecular docking calculated by PyMol 1.8.6 software showed the binding energies of dobinin K with wild type of *Pf*NDH2 and *Pf*DHODH protein were −7.0 kcal/mol and −8.0 kcal/mol, respectively. As shown in *Pf*NDH2 results (Table 1), dobinin K fits the pocket well through hydrophobic interactions with adjacent residues including PRO71, VAL148 and THR78, hydrogen bond interactions with ARG72, and salt bridges interactions with SER48. To further verify the effect of the above residues on the binding ability of dobinin K and *Pf*NDH2, PyMol 1.8.6 software was used to knock out the corresponding residues and to calculate the binding energies of dobinin K with the mutant *Pf*NDH2 protein (Table 1). The results showed that the binding energy of dobinin K with the mutant proteins after theoretical knockout of VAL148, THR78, ARG72, and SER48 were increased, which demonstrated their binding stabilities were decreased (Table 1, Figure 9A). And the mutant *Pf*NDH2 with SER48 theoretical knockout had the lowest binding stability to dobinin K. As shown in *Pf*DHODH results (Table 2), dobinin K fits the pocket well through hydrophobic interactions with adjacent residues TYR528 and LEU481, hydrogen bond interactions with residues include LYS429, SER477, LYS229 and THR459. After the theoretical knockout of LEU481, LYS229, and THR459, the binding stability between dobinin K with the mutant *Pf*DHODH were decreased (Table 2, Figure 9B). The mutant protein with THR459 theoretical knockout had the lowest binding stability to dobinin K.

## 3. Discussion

In this study, a novel sesquiterpenoid dobinin K was confirmed as a potential lead compound against malaria, although the anti-malaria ability of dobinin K was weaker than chloroquine and artemisinin (Supplementary Materials Table S1). It was not only effective for the chloroquine-sensitive strain 3D7 but also for the artemisinin-resistant strain 803 and the chloroquine-resistant Dd2 strain. At the same time, the combination of dobinin K and chloroquine could synergistically inhibit the growth of Dd2. The effect of dobinin K on the 3D7 was time-dependent, and persistent even after the withdrawal of dobinin K. The inhibitory effect of dobinin K was also stage-specific; that is, dobinin K showed an obvious effect on the trophozoite and schizont, which were the vigorous life activity stages.

Transcriptomics was applied to predict the target of dobinin K. The analysis showed the expression of ferredoxin-NADP reductase, dihydrolipoyl dehydrogenase, and glucose-6-phosphate dehydrogenase-6-phosphogluconolactonase significantly decreased after dobinin K treatment. Since all these predicted targets were involved in the redox system, we hypothesized that dobinin K could affect the ROS level in the parasite to execute plasmodial action during erythrocytic stages. The oxidative stress produced by ROS will then weaken the antioxidant defense system of *P. falciparum* like the NADPH-dependent thioredoxin and glutathione systems, and induce NADPH oxidation, followed decrease of NADPH/NADP^+^ ratio [16,17,18,19]. The pentose phosphate pathway is the main source of NADPH in *Plasmodium*, herein the decrease of NADPH may be due to the down-regulation of the pentose phosphate pathway, which was predicted by KEGG analysis. Then, following the prolongation of drug treatment, *P. falciparum* loses the ability to regulate the redox system, which eventually leads to apoptosis of the parasite’s cell [20,21]. Therefore, we focused on the redox system to study the acting mechanism of dobinin K.

For confirmation of our hypothesis, the ROS level in the parasite was first examined. With the treatment of dobinin K, ROS levels enhanced in a concentration-dependent manner, in detail, 50 μM and 200 μM dobinin K increased intracellular ROS levels by 9-fold and 39-fold in *P. falciparum*, and the effect of the compound at all concentrations were better than the positive control. The dramatically elevated ROS levels will induce an imbalance of the redox system of *P. falciparum*, damage nucleic acids, proteins, and membrane lipids, and eventually lead to the apoptosis of *P. falciparum* [22], which was confirmed by different methods including Giemsa-staining, Hoechst 33258 dying, and TUNEL staining. After incubation of dobinin K, the parasite showed obvious apoptotic morphology, such as deep nuclear staining, chromatin condensation, and concentration-dependent DNA fragmentation as the document mentioned [23].

Mitochondria of *P. falciparum* play a crucial role in stimulation-induced apoptosis [11]. Unlike mammals, the energy production of *P. falciparum* does not depend on mitochondrial ETC, but instead on cytoplasmic glycolysis [12]. The main function of the *P. falciparum* mitochondria appears to mediate the de novo biosynthesis of pyrimidine since *P. falciparum* lacks of pyrimidine salvage pathway [15]. The disruption of mitochondrial ETC leads to the loss of all pyrimidine sources of *P. falciparum*. Thus, this organelle becomes a potential target for antimalarial drugs. In our study, dobinin K seemed to be an antimitochondrial compound because of its ability to reduce oxygen consumption of *P. falciparum* mitochondria ETC and disrupt mitochondria membrane potential. When mitochondria are damaged due to oxidative stress by dobinin K, the loss of mitochondrial membrane potential and the cessation of metabolic activity promote the apoptosis of *P. falciparum* [24]. The ETC of *P. falciparum* is known to have five dehydrogenases, namely NDH2, SDH, MQO, DHODH, and G3PDH [11]. Here, dobinin K selectively inhibited the activities of NDH2 and DHODH in a concentration-dependent manner. *Pf*NDH2 is a principal electron donor to the ETC and participates in the redox reaction of NADH oxidation with subsequent production of ubiquinone [25,26]. Electron transfer from NADH to ubiquinone has been shown to maintain the oxidized NADH pool, which is used in metabolic processes such as redox homeostasis regulation and glycolysis [26,27]. *Pf*DHODH is not only the enzyme involved in the redox reaction from dihydroorotate to orotate, but also participates in the de novo pyrimidine biosynthesis [15,28,29]. The effects of dobinin K on *Pf*NDH2 and *Pf*DHODH partly explained its disruption of the potential across the mitochondrial inner membrane. Molecular docking was further applied to simulate the interaction between dobinin K and *Pf*NDH2 or *Pf*DHODH. The results of molecular docking showed that dobinin K has good binding stabilities with *Pf*NDH2 and *Pf*DHODH. The SER48 is the key residue to the interaction of dobinin K and *Pf*NDH2, and the THR459 is that to the binding of dobinin K with *Pf*DHODH. It should be noted that resistance of *Plasmodia* to atovaquone, an antimitochondrial compound that targets to cytochrome bc1 complex [30], has occurred [31,32]. Therefore, the act of new antimitochondrial compounds against resistant Plasmodia needs to be evaluated. Fortunately, dobinin K displayed extensive inhibitory power on the chloroquine-resistant strain and the artemisinin-resistant strain. The fact that dobinin K is effective against the above classic antimalarials-resistant *Plasmodium* may be due to the different acting mechanisms between them. Chloroquine, can accumulate in the acidic food vacuole of *Plasmodium*, then interfere with the process of conversion of heme into nontoxic hemozoin crystals, lead to the accumulation of toxic heme within the parasite, and finally ultimately result in parasite’s death [33]. In the chloroquine-resistant *Plasmodium*, a mutation of *Pf*CRT protein on the food vacuole occurs to increase the efflux of chloroquine from the food vacuole [34]. Artemisinin contains an endoperoxide bridge, which is the core structure to directly produce ROS with the influence of iron ions derived from heme [35]. However, in some artemisinin-resistant *Plasmodia*, the parasites can extend their dormancy period to evade artemisinin (whose half-life is only 2–4 h) induced damage [36]. Here, dobinin K produces ROS indirectly derived from the disruption of *P.* falciparum mitochondria, which is unlike the mechanism of chloroquine and artemisinin. Thus, dobinin K can inhibit the growth of chloroquine- and artemisinin-resistant *Plasmodium.*

Up until now, natural products are still a major resource of antimalaria drugs. Dobinin K is a novel chemical obtained from *Dobinea delavayi*, whose antiplasmodial activity has not been reported before. We hope dobinin K could be a reserve antimalaria lead compound, and a series of compounds that come from dobinin K can be applied against resistant Plasmodia.

## 4. Materials and Methods

### 4.1. Chemicals and Reagents

Cell culture medium RPMI Medium 1640 (Lot No. 2503686) and Albumax II (Lot No. 2605576) were Gibco (New York, NY, USA) products. Cell mitochondria isolation kit (Lot No. 033023230511), mitochondrial membrane potential assay kit (Lot No. 030822220726), and ROS assay kit (Lot No. 051123230719) were purchased from Beyotime Biotechnology (Shanghai, China). The oxygen consumption assay kit (Lot No.BB22121) was purchased from Bestbio (Shanghai, China). Ubiquinone (Lot No. M08IS214249) and 2,6-Dichloroindophenol (Lot No. A18IS212811) were purchased from Yuanye Bio-Technology Co., Ltd., (Shanghai, China). NADH (Lot No. C23HY 03182602P) was purchased from Harveyblo Gene Technology Co., Ltd., (Beijing, China).

Complete media (CM) contained RPMI-1640 medium, 25 mM HEPES, 370 μM hypoxanthine and 36 µM gentamycin, 0.2% NaHCO3, and 0.5% Albumax II. Incomplete media (ICM) was CM without Albumax II.

### 4.2. P. falciparum Culture

The *P. falciparum* strains (3D7, Dd2, 803) were kindly provided by the Shanghai Institute of Immunity and Infection, Chinese Academy of Sciences (Shanghai, China). These parasites were cultivated in O+ human red blood cells (RBCs) using CM at 37 °C and 5% CO_2_. When the infection rate is less than 1%, the culture medium is changed every 24 h. Synchronization of the *P. falciparum* culture to the ring stage was obtained using 5% sorbitol. The percentage of infected RBCs (iRBCs) was determined by a count of 1000 cells in a thin blood smear stained with Giemsa and viewed under a light microscope. This protocol has been approved by the Institutional Ethical Committee of Dali University (No: 201908-01).

### 4.3. Concentration-Dependent Parasite Inhibition Assay

Dobinin K was dissolved in DMSO and diluted to different concentrations with a culture medium. A synchronous ring stage culture (3D7, Dd2, or 803) with 1.0% parasitemia and 2% hematocrit was incubated in a 96-well plate with dobinin K (50 to 200 μM) at 37 °C for 72 h. Then, 100 μL red blood cell lysis buffer containing SYBR Green I was added to each well, and the culture was incubated in the dark for 2 h. The inhibition rate of dobinin K was determined by the measurement of the SYBR Green I fluorescence using a fluorescence enzyme spectrometer (Varioskan LUX, Thermo, Waltham, MA, USA) at wavelengths 490 nm and 530 nm for excitation and emission, respectively. The antiplasmodial activity of dobinin K was represented as IC_50_ calculated from the concentration-response curve by nonlinear regression analysis. Triplicate assays were performed in parallel.

### 4.4. Antiplasmodial Activity Determination of the Combination with Dobinin K and Chloroquine against Dd2 Strain

Four concentrations of dobinin K (0, 50, 80, and 130 μM) and four concentrations of chloroquine (0, 57, 74, and 96 nM) were chosen to evaluate their combination effect. The inhibition rates of every concentration of dobinin K alone, chloroquine alone, and their pairwise combination were measured. Online software SynergyFinder 3.0 (https://synergyfinder.fimm.fi/, accessed on 29 October 2023) was applied to analyze the combination response data, and the Zero Interaction Potency (ZIP) model was used to calculate the synergy scores. The interaction between two drugs is determined by the score. That is, score < −10 represents antagonism, −10 < score < 10 represents addition, and score > 10 represents synergism.

### 4.5. Time-Dependent Parasite Inhibition Assay

Asynchronous parasites (2% infection rate, 2% hematocrit) were treated with 100 μM dobinin K. At 3, 6, 12, and 24 h after treatment, the infection rate was examined with the Giemsa staining followed the washout of the drug with ICM. Then, the parasite was diluted with new RBCs at a ratio of 1:40 and for an additional 96 h of culture. Parasitemia of each group was re-examined as the above stain method. DMSO was used as a vehicle control. Triplicate assays were performed in parallel.

### 4.6. Stage-Specific Parasite Inhibition Assay

Highly synchronized parasites were cultured in 24-well plates (2% infection rate, 2% hematocrit) and incubated with 100 μM dobinin K on different parasite stages for 12 h. After that, the culture was washed 3 times with ICM to remove the compound and continued to cultivate until 50 hpi (hours post-invasion). Then, parasitemia was examined by Giemsa staining and morphological analysis was performed by a light microscope (EX30, Sunny Optical Technology Co., Ltd., Ningbo, China). The rest parasite was diluted 1:40 with new RBCs for an additional 96 h (146 hpi) of culture to explore the long-term effect of dobinin K. DMSO was used as a vehicle control. Triplicate assays were performed in parallel.

### 4.7. Isolation of P. falciparum and Mitochondria

The highly synchronized trophozoite-stage *P. falciparum* cultures were transferred to a tube for centrifugation (600× *g*, 4 °C, 5 min), and iRBCs were collected. These iRBCs were incubated with 0.075% (*w*/*v*) saponin for 10 min at 0 °C. The precipitate (that is *P. falciparum*) was collected from the mixture by centrifugation (90× *g*, 4 °C, 5 min). Then the *P. falciparum* was treated with a mitochondrial extract solution at 4 °C for 15 min. The cell membrane was destroyed by homogenate (50 Hz for 60 s) and large cell fragments were removed by centrifugation (600× *g*, 4 °C, 10 min). The supernatant was transferred to a tube for centrifugation (11,000× *g*, 4 °C, 10 min) and the precipitate was collected as *Plasmodium* mitochondria. The isolated mitochondria were kept in a storage solution in the kit at 4 °C.

### 4.8. Determination of ROS Production

Intracellular ROS production was determined by the DCFH-DA method. DCFH-DA is a non-fluorescent dye and can freely cross the cell membrane. In the cytoplasm, DCFH-DA is hydrolyzed to DCFH, which is reacted with intracellular ROS to produce fluorescent. The isolated parasite was suspended in the culture media containing DCFH-DA (10 μM), transferred to the 96-well plate, and incubated for 30 min. Then, dobinin K was added to the corresponding well and quickly placed into the preheated fluorescent enzyme-labeled instrument. The excitation wavelength of 488 nm and the emission wavelength of 525 nm were used to detect the intensity of fluorescence by a fluorescence enzyme spectrometer. During a total of 60 min, the interval between each measurement is 3 min. Triplicate assays were performed in parallel.

### 4.9. Detection of Oxygen Consumption

The isolated mitochondria were diluted in 100 μL mitochondrial storage solution, and dobinin K was added so that the final concentration was 100 μM. DMSO was used as a vehicle control group. An amount of 10 μM FCCP or 1 μM antimycin A was applied as the positive control. After the addition of 4 μL R01 fluorescent dye (the oxygen probe), 100 μL of oxygen-blocking solution was immediately added. The mitochondrial oxygen consumption was measured at 37 °C in the fluorescence enzyme spectrometer once every 2 min for 60 min and then the oxygen consumption curve was drawn.

### 4.10. Measurement of Mitochondrial Membrane Potential

The mitochondrial transmembrane potential was analyzed with the JC-1 fluorescent dye. When the mitochondrial function is normal, its membrane potential is high, and JC-1 accumulates in the matrix of the mitochondria to form a polymer (J-aggregates), which can produce red fluorescence with a maximum excitation wavelength of 590 nm. But when mitochondria are damaged, their membrane potential becomes low, and JC-1 cannot gather in the matrix of mitochondria. At this time, JC-1 is a monomer that can produce green fluorescence with a maximum excitation wavelength of 530 nm. The prepared JC-1 dyeing solution was diluted 5 times with buffer to prepare a working solution. Next, 10 μL purified mitochondria were added to 90 μL JC-1 working solution, and dobinin K was added at concentrations of 200 μM, 100 μM, and 50 μM. After the mixture, JC-1 fluorescent at 530 nm and 590 nm was measured by the fluorescence enzyme spectrometer. CCCP was used as the positive control. The working solution containing dobinin K without mitochondria was applied as the blank control.

### 4.11. Hoechst 33258 Staining Assay

The nuclear morphology of cell apoptosis was observed by Hoechst 33258 staining. After incubation with different concentrations of dobinin K for 12 h, *P. falciparum* was isolated and then fixed with 4% (*w*/*v*) paraformaldehyde for 15 min at room temperature. Later, these parasites were washed 3 times with PBS and stained with Hoechst 33258 solution (5 μg/mL) for 20 min at 37 °C. Then, the images were captured and observed using the high-content imaging analysis system (Operetta Cl, Revvity, Waltham, MA, USA) with an excitation wavelength of 352 nm.

### 4.12. TUNEL Assay

TUNEL kit (Servicebio, Wuhan, China) was used to probe DNA fragmentation of cell apoptosis. Drug-treated *P. falciparum* was isolated, fixed with 4% paraformaldehyde, and incubated with tetramethyl rhodamine-labeled dUTP buffer at 37 °C for 1 h. After washout, these parasites were stained with DAPI solution for 5 min at room temperature. Then, the images were captured and observed using the fluorescence microscope (BXS3, Olympus, Tokyo, Japan). The excitation wavelength of 551 nm and the emission wavelength of 575 nm were used. The fluorescence intensity of the image was calculated using Image-J 1.0.8.345.

### 4.13. Measurement of Enzyme Activities in the ETC

The ETC of Plasmodia is composed of five dehydrogenases, including dihydroorotate dehydrogenase (DHODH), malate: quinone oxidoreductase (MQO), glycerol-3-phosphate dehydrogenase (G3PDH), succinate dehydrogenase (SDH), and type-II NADH dehydrogenase (NDH2). Among these dehydrogenases, all the reduction products of DHODH, MQO, G3PDH, and SQR use 2,6-dichlorophenolindophenol (DCIP) as an electron acceptor, and their activities were determined by monitoring the absorbance change of DCIP using a microplate reader (Varioskan LUX, Thermo, Waltham, MA, USA) at 600 nm (reflecting DCIP reduction). The reduction product of NDH2 is NADH which has its absorbance. Thus, the activity of NDH2 could be determined by monitoring the absorbance change of NADH at 340 nm (reflecting NADH reduction). The procedure for measurement of enzyme activities was as follows: (1) Preparation of reaction buffer system. The system for DHODH, MQO, and G3PDH contains 45 µM DCIP, 100 µM ubiquinone, and 2 mM antimycin A in 30 mM Tris-HCl, pH 8.0. The system for SQR contains the same reagent as above in 50 mM potassium phosphate, pH 8.0. The system for NDH2 contains 100 µM ubiquinone and 2 mM antimycin A in 50 mM potassium phosphate, pH 8.0. (2) Isolated *P. falciparum* mitochondria were incubated with a dobinin K-containing reaction buffer system. (3) 500 µM dihydroorotate (substrate of DHODH), 10 mM malate (substrate of MQO), 500 µM glycerol-3-phosphate (substrate of G3PDH), 10 mM succinate (substrate of SQR), or 20 µM NADH (substrate of NDH2) was added to the system to initiate the reaction of reduction, respectively. Then, the activities of these dehydrogenases were measured by the absorbance of DCIP (5 min after reaction) or NADH (1 min after reaction).

### 4.14. Transcriptomic Analysis

Synchronized parasites (17–29 hpi) were cultured in flasks and incubated with 73 μM dobinin K for 12 h. Isolated parasites were collected and the total RNA were extracted with the TRIzol reagent (Tiangen, Beijing, China). The RNA samples were frozen and sent to the Applied Protein Technology company (Shanghai, China) for transcriptome sequencing and analysis [37].

### 4.15. Molecular Docking Study

The 2D structure of dobinin K is imported into Chem 3D ultra 20.0 software for converting SDF format to Mol 2 format (3D structure). The *Pf*NDH2 protein (PDB ID: 5jwc) and *Pf*DHODH protein (PDB ID: 3i68) were obtained by using the PDB database (https://www.rcsb.org/, accessed on 4 April 2024). Structures of target proteins and dobinin K were pretreated by Auto Dock Tools 1.5.6. The docking range was set at the position of the original ligand. Molecular docking between target proteins and dobinin K was performed with the Lamarckian genetic algorithm by Auto Dock Vinav.1.2.0 software. The semi-flexible docking method was applied to obtain the docking-binding free energy. When the binding energy score < −4.25, the binding power between the test molecule and the protein is considered to exist. A score of less than −5.0 indicates a relatively high binding affinity between the molecule and protein. After docking, the sites with relatively high binding affinity were obtained, non-covalent interactions between protein and dobinin K were obtained by the protein-ligand interaction profiler, and the amino acid residues that bind to dobinin K were identified. Then, PyMol software was used to display molecular docking conformation. For exploration of amino acid residues’ effects on the binding ability of dobinin K and target proteins, the residue that binds to dobinin K was knockout by PyMol software, and the lowest binding conformation between dobinin K and mutant protein with removed amino acid residues was obtained by autodock1.5.6 software.

### 4.16. Statistical Analysis

Data were shown as mean ± standard deviation (SD) and analyzed by SPSS 20.0 software. Differences between each group were calculated using the one-way analysis of variance (ANOVA) followed by Tukey’s post hoc test. *p* < 0.05 was regarded to be statistically significant.

## Figures and Tables

**Table 1:** Molecular docking results of dobinin K and *Pf*NDH2.

Residue No.	Amino Acids	Force of Interaction	Binding Energies after Mutated Residues
71	PRO	Hydrophobic Interactions	−7.3
148	VAL	Hydrophobic Interactions	−6.9
78	THR	Hydrophobic Interactions	−6.8
72	ARG	Salt Bridges	−6.7
48	SER	Hydrogen Bonds	−6.6

**Table 2:** Molecular docking results of dobinin K and *Pf*DHODH.

Residue No.	Amino Acids	Force of Interaction	Binding Energies after Mutated Residues
528	TYR	Hydrophobic Interactions	−8.0
481	LEU	Hydrophobic Interactions	−7.9
429	LYS	Hydrogen Bonds	−8.6
477	SER	Hydrogen Bonds	−8.0
229	LYS	Hydrogen Bonds	−7.7
459	THR	Hydrogen Bonds	−7.5

## Data Availability

The data presented in this study are available upon request from the corresponding author.

## Supplementary Materials

The following supporting information can be downloaded at: https://www.mdpi.com/article/10.3390/molecules29194759/s1, Table S1: supplementary Table 1.
